# 

**Published:** 1858-11

**Authors:** 


### LIST OF COLLABORATORS.

S. G. Armor, M.D., late Professor of Pathology and Clinical Medicine, Missouri Med. College.
John L. Atlee, M.D., late President Pennsylvania State Medical Society.
Walter F. Atlee, M.D., Philadelphia.
Robert G. Barclay, M.D., Jerusalem, Syria.
John Bell, M.D., Philadelphia, formerly Professor of Medicine, Medical College of Ohio.
T. S. Bell, M.D., Professor of Medicine, University of Louisville.
S. M. Bemiss, M.D., Professor of Clinical Medicine, University of Louisville.
L. P. Blackburn, M.D., New Orleans, La.
George C. Blackie, M.D., Professor of Botany, University of Nashville.
G. C. Blackman, M.D., Professor of Surgery, Medical College of Ohio.
J. L. Bobbs, M.D., formerly Professor of Anatomy, Indianapolis.
W. M. Boling, M.D., Montgomery, Ala., formerly Professor of Midwifery, Transylvania University, Lexington, Ky.
W. S. Bowen, M.D., New York.
N. Bozeman, M.D., Montgomery, Ala.
J. T. Bradford, M.D., Augusta, Ky.
John H. Brinton, M.D., Lecturer on Operative Surgery, Philadelphia.
W. S. Chipley, M.D., Professor of Medicine, Transylvania University.
A. Clapp, M.D., New Albany, Ia.
D. B. Cliffe, M.D., Franklin, Tenn.
J. Da Costa, M.D., Lecturer on the Practice of Medicine at the Philad. Med. Association.
Samuel H. Dickson, M.D., Professor of Medicine, Jefferson Medical College.
M. Dupierris, M.D., Havana, Cuba.
Richard J. Dunglison, M.D., Philadelphia.
W. F. Edgar, M.D., United States Army.
S. C. Farrar, M.D., Jackson, Miss.
C. S. Fenner, M.D., Memphis, Tenn.
Austin Flint, M.D., Professor of Clinical Medicine, Auscultation and Percussion, New Orleans School of Medicine.
Charles Frick, M.D., Baltimore.
W. Y. Gadbury, M.D., Benton, Miss.
O. C. Gibbs, M.D., Chautauque County, New York.
Dr. J. P. Hall, Glasgow, Ky.
William A. Hammond, M.D., United States Army.

JOHN HARDIN, M.D., Professor of Obstetrics, Kentucky School of Medicine, Louisville.

EDWARD HARTSHORNE, M.D., One of the Surgeons to Wills Hospital for Diseases of the Eye, Philadelphia.

E. B. HASKINS, M.D., Professor of Chemistry, Shelby Medical College, Nashville, Tenn.

C. A. HENTZ, M.D., Marianna, Florida.

ADDINELL HEWSON, M.D., One of the Surgeons to Wills Hospital for Diseases of the Eye, Philadelphia.

SAMUEL L. HOLLINGSWORTH, M.D., late Editor of the Medical Examiner, Philadelphia.

WILSON JEWELL, M.D., Philadelphia, President of the Quarantine and Sanitary Convention.

WM. V. KEATING, M.D., Lecturer on Obstetrics and the Diseases of Women, at the Philadelphia Medical Association.

JOHN G. KERR, M.D., Canton, China.

G. A. KUNKLER, M.D., Madison, Ia.

BEN. LOGAN, M.D., Shreveport, La.

A. LOPEZ, M.D., Mobile, Ala., formerly President of the Alabama State Medical Society.

J. AITKEN MEIGS, M.D., Professor of the Institutes of Medicine in the Philadelphia College of Medicine.

S. WEIR MITCHELL, M.D., Lecturer on Physiology at the Philad. Med. Association.

GEORGE R. MOREHOUSE, M.D., Philadelphia.

B. I. RAPHAEL, M.D., New York.

J. C. REEVE, M.D., Dayton, Ohio.

L. C. RIVES, M.D., formerly Professor of Midwifery, Medical College of Ohio.

J. LAWRENCE SMITH, M.D., Professor of Chemistry, University of Louisville.

W. C. SNEED, M.D., Frankfort, Ky., late President Kentucky State Medical Society.

C. H. SPILMAN, M.D., Harrodsburg, Ky., late President of Kentucky State Medical Society.

GEO. SUTTON, M.D., Aurora, Ia.

W. L. SUTTON, M.D., Georgetown, Ky., formerly President Kentucky State Medical Society.

HORATIO R. STORER, M.D., formerly one of the Physicians to the Boston Lying-in Hospital, Boston, Mass.

S. THOMPSON, M.D., Albion, Ill., late President of the Illinois State Medical Society.

E. WILLIAMS, M.D., Cincinnati, Ohio.

### Bi-Monthly Bibliographical Record.

### AMERICAN.

Diseases of the Urinary Organs; a Compendium of their Diagnosis, Pathology, and Treatment. By William Wallace Morland, M.D., etc. 8vo., pp. 579. Philadelphia: Blanchard & Lea, 1858. (From the publishers.)

Concentrated Organic Medicines; being a Practical Exposition of the Therapeutic Properties and Clinical Employment of the Combined Proximate Medicinal Constituents of Indigenous and Foreign Plants. 8vo., pp. 432. B. Keith & Co.: New York, 1858. (From the publishers.)

Selections from Favorite Prescriptions of Living American Practitioners. By Horace Green, M.D., L.L.D., etc. 8vo., pp. 206. Wiley & Halsted: New York, 1858. (From the publishers.)

Induced Abortion on Account of Extreme Narrowness of the Pelvis. Prize Essay of the Medical Faculty at Tübingen. By Ferd. Rattenman, M.D., etc. Pamphlet, pp. 51. Philadelphia, 1858. (From the author.)

The Physician's Visiting List, Diary, and Book of Engagements, for 1859. Lindsay & Blakiston: Philadelphia. (From the publishers.)

Catalogue of the Surgical and Pathological Museum of Valentine Mott, M.D., etc., and of his son, Alex. B. Mott, M.D., etc. New York, 1858. (From the author.)

Physiology, Pathology, and Therapeutics of Muscular Exercise. A paper read before the Cook County Medical Society, and published at their request. By W. H. Byford, M.D. Chicago, 1858. (From the author.)

Sixth Annual Report of the Superintendent of Hamilton County Lunatic Asylum, for the year ending June 7th, 1858. Pamphlet, pp. 21. Cincinnati.

### FOREIGN.

Lectures on the Principles and Practice of Physic. By Thomas Watson, M.D., F.R.C.P., etc. A new American, from the last revised and enlarged London edition. Edited, with additions, by Dr. Francis Condie, M.D., etc. 8vo., pp. 1224. Philadelphia: Blanchard & Lea, 1858. (From the publishers.)

A System of Human Anatomy, General and Special. By Erasmus Wilson, F.R.S., etc. A new and improved American, from an enlarged London edition. Edited by William Gobrecht, M.D., etc. 8vo., pp. 616. Philadelphia: Blanchard & Lea, 1858. (From the publishers.)

Lectures on the Diseases of Women. By Charles West, M.D., etc. etc. 8vo., part second, pp. 181. Philadelphia: Blanchard & Lea, 1858. (From the publishers.)

A Course of Lectures on Obstetrics; delivered at St. Mary's Hospital, London. By Wm. Tyler Smith, M.D., etc. With an Introductory Lecture on the Art of Midwifery, and copious practical Annotations. By A. K. Gardner, A.M., M.D., etc. 8vo., pp. 760. R. M. DeWitt: New York, 1858. (From the publishers and editor.)

Anatomy, Descriptive and Surgical. By Henry Gray, F.R.S., Lecturer on Anatomy at St. George's Hospital. Royal 8vo., pp. 750. John W. Parker & Son: London, 1858.

An Inaugural Dissertation on Strychnia; presented to the Medical Faculty of McGill College. By Alex P. Reid. Pamphlet, pp. 39. Montreal, 1858.

The Uræmic Convulsions of Pregnancy, Parturition, and Childbed. By Carl R. Braun, Professor of Midwifery, Vienna. Translated from the German, with Notes, by J. Matthews Duncan, F.R.C.P.E., etc. 12mo., pp. 182. New York: S. S. & W. Wood, 1858. (From the publishers.)

De La Fièvre Puerpérale devant l'Académie Impériale de Médecine de Paris, et des principes du vitalisme hippocratique appliqué à la solution de cette question. Par le Docteur T. C. Auber. 8vo., pp. 107. Germer Ballière: Paris, 1858.

On the Nature, Causes, Statistics, and Treatment of Erysipelas. By Peter Hinckes Bird, F.R.C.S. London: John Churchill.

A Life of Linnæus. By Miss Brightwell, of Norwich. John Van Voorst: London, 1858.

Stricture of the Urethra; its Pathology and Treatment. By Henry Thompson, F.R.C.S., etc. London: John Churchill, 1858.

Du Prognostic de l'Épilepsie et du Traitement de cette Maladie par le Valérianate d'Atropine. Par le Dr. Michléa. Labé: Paris, 1858.

On Medicine and Medical Education. By W. T. Gardner, M.D., F.R.C.P.E., etc. 12mo., pp. 130. Edinburgh: Sutherland & Knox, 1858.

Sur une Functien peu connue du Pancréas, la Digestion des Aliments Azotés; Expériences parallèles sur la Digestion Gastrique et Intestinale; Inductions Cliniques. Par le Docteur Corvisart. 8vo., pp. 123. Victor Masson: Paris, 1858.

Electro-Chemistry, with Positive Results and Notes. By Charles Chalmers, late of Merchiston Academy. pp. 100. London, 1858.

### INDEX.

Abbot, S. J., M.D., on oxide of zinc in night-sweats, 1084.
Abdominal viscera, cancer of, 101.
Abdomen, fibro-fatty tumor of, 517.
incised wound of, 1070.
Abscess of liver, 169.
metastatic, of liver, 1075.
of iliac fossa, new mode of treatment of, 536.
tubercular of kidney, 1072.
of ovary, 705.
of brain, 873, 1074.
lumbar, 539.
Académie de Médecine, prizes for 1857, 398.
Acetic acid, dilute, in scarlatina, 1088.
Accidents of Knighthood, 587.
Aconite, use of in neuralgia, 934.
Acton, Wm., treatise on prostitution by, review of, 977.
Adams, Robt., M.D., treatise on rheumatic gout, &c., by, notice of, 850.
Addison's disease, case of, 376.
Advertisements, quack, 179.
African edibles, 391.
Albumen, gum, &c., physiological effects of, 126.
Alimentary canal, treatise on diseases of, by Habershon, review of, 236.
Allopathy, 1147.
Amenorrhœa, electro-magnetism in, 1085.
American Medical Association, notice of proceedings of, 775.
Ammonia, valerianate of, in neuralgia, 570, 934.
Amputation at hip-joint, 567.
of neck of uterus, 951.
new method of, by Maisonneuve, 947.
of arm, 1069.
Anæsthesia universalis peripherica, 1106.
Anæsthetics, use of in convulsions, 937.
Anal fistula, its relation to phthisis, 371.
Anatomy, treatise on, in Chinese, by Hobson, notice of, 43.
pathological, treatise on, by Gross, review of, 1.
by Andral, review of, 1.
by Broussais, review of, 1.
Anatomy, pathological, treatise on, by Cruveilhier, review of, 1.
by Horner, review of, 1.
by Hasse, review of, 1.
by Otto, review of, 1.
by Rokitansky, review of, 1
by Vogel, review of, 1.
by Wedl, review of, 1.
of inflammations, treatise on, by Gendrin, review of, 1.
Anatomy and Physiology, report on, by Mitchell, 105.
Anderson, T. M., M.D., case of ileus by, 176.
Andral, G., M.D., treatise on pathological anatomy by, review of, 1.
Aneurism of aorta, 509, 532, 724.
of innominata, 511.
of external iliac, 549.
femoral, treated by manipulation and compression, 544.
by ligature, 547, 550.
popliteal, treated by compression, 545, 546, 579.
Angell, C., M.D., ligature of carotid artery by, 157, 350.
Animal signatures, 398.
Ankle-joint, excision of, 580.
Antidote to poison of rattlesnake, 385.
to strychnia, 1098, 1099, 1102.
to chloroform, 1099.
to many poisons, 1099.
Antimony, tartrate, in colic, 945.
as an enema, 1096.
Anus, imperforate, 575.
Aorta, aneurism of arch of, 509, 532, 724.
Aortic valves, perforation of, 870.
Apoplexy, infantile, 70.
after trephining, 320.
effect of on the intellect, 351.
of lung, 317.
of retina, 730.
Appendix vermiformis, perforation of, 360.
Aran, M., on laudanum dressings, 967.
Armor, S. G., M.D., resignation of, 780.
Army, appointments to medical staff of, 970.
Arsenic, cure of cholera by, 165.

Arsenic in skin diseases, 1096.
Artery, dorsalis penis, ligature of, 155.
carotid, ligature of, 157, 159, 350, 541, 543.
external iliac, ligature of, 547, 550, 551.
common iliac, ligature of, 549.
femoral, ligature of, 550.
innominate, aneurism of, 511.
meningeal, dilatation of, 726, 1072.
Arteries, ossification of, in gangrene, 507.
atheromatous deposit in, 515.
Arthritis, chronic, Treatise on, by Adams, notice of, 850.
rheumatic, cases of, 713.
Asclepias tuberosa, virtues of, 945.
Syrica in intermittent fever, 1090.
Ass's milk in atrophy of bones, 505.
Astragalus, necrosis and discharge of, 712.
Asthma, influence of atmospheric changes on, 372.
iodide of potassium in, 1084.
treatment and clinical history of, 1134.
Atheromatous deposit in arteries, 515.
Atlee, J. L., M.D., on the écraseur in ovariotomy, 698.
Atrophy, yellow, of liver, 1108.
Auricle, right, functions of, 378.
ossific deposit in walls of, 532.
Ayres, H. P., M.D., on hydro-cerebral hernia, 73.
Ayres, D., M.D., on dislocation of cervical vertebræ, 747.

Bache, Franklin, M.D., Dispensatory by, notice of, 465.
Baker, C. H., M.D., reduction of dislocated femur by, 750.
Bamberger, Dr., treatise on pathology by, review of, 414.
on yellow atrophy of liver, 1108.
Barclay, A. W., M.D., treatise on medical diagnosis by, review of, 401.
Barker, J. F., M.D., on veratrum viride, 926.
Baudens, M., necrological notice of, 591.
Becquerel and Rodier, treatise on pathological chemistry by, notice of, 263.
Bell, John, M.D., report on materia medica by, 910, 1079.
Bell, Dr. J., on effects of cannabis indica, 942.
Belladonna, use of, to arrest secretion of milk, 946.
Bennett, J. H., M.D. clinical lectures on medicine by, review of, 618.
Benson, Dr., appointment of, 968.
Berard, Prof., on extirpation of pancreas, 765.
Bernard, Ch., on engorgement of cervix uteri, 952.
on use of carbonic acid gas, 966.
Bibron's antidote for rattlesnake poison, 385, 1100.
Bile, physiology of, 128, 379.
Binz, Dr., on universal anæsthesia, 1106.
Biological Society of Philadelphia, notice of, 193.
Birkett, Mr., case of popliteal aneurism by, 579.
Bird, Golding, M.D., treatise on urinary deposits, review of, 785.
Black, C., M.D., on arsenic in cholera, 165.
Blackman, G. C., M.D., on ligature of arteries, &c., 543, 544, 558, 569, 750.
Bladder, stone in, 753, 754, 756.
lead-pencil removed from, 754.
gangrene of, 96.
chronic inflammation of, 1072.
removal of stones from, 1127.
Blood, properties and uses of, 135, 761.
circulation of, 378.
injection of urea into, 287.
Bœrstler, Dr. G. W., on veratrum viride, 916.
Bones, eburnation of, 312.
atrophy of, 505.
scooping of, in caries, &c., 948.
syphilitic disease of, 949.
excision of, in British hospitals, 955.
Bonsall, T. C., M.D., on iodide of potassium in syphilis, 1080.
Bony tumor of scrotum, 79, 100.
Botany, works on, by Gray, review of, 22, 265.
Bowditch, H. J., M.D., on paracentesis, &c., 368, 753.
Bowling, W. K., M.D., on creasote in cholera, 1082.
Bowels, intussusception of, 359.
Boylston prize essay, announcement of, 194.
Brain, injury of, 75.
chronic abscess of, 873.
chemical composition of, 1104.
influence of spinal cord on, 1104.
serous sac of middle ventricle, 1105.
Brannock and Black, Drs., on virtues of sulphate of cinchona, 1090.
Braun, Prof. C., on hydrorrhœa of uterus, 950.
treatise on uræmic convulsions, &c. by, review of, 1020.
Breant prize not awarded, 971.
Breast, malignant tumor near, 580.
Bright's disease, 327, 727.
Brinton, Wm., M.D., treatise on ulcer of the stomach by, review of, 1028.
Brodie, Sir Benjamin, nomination of, 971.

Brodie, R. T., M.D., reduction of dislocated femur by, 751.
Brokaw, F. V. L., M.D., on abscess of liver, 169.
Bronchial tubes, ulceration of, 868.
Bronzed skin with cirrhosis of liver, 377.
Broussais, F. J. V., treatise on phlegmasiæ, review of, 1.
Brown-Séquard on properties and uses of blood, 135, 761.
journal of physiology by, notice of, 193, 586.
researches on physiology and pathology, review of, 222.
on epilepsy, review of, 222.
on influences of spinal cord on brain, 1104.
on measurement of sensibility in morbid state, 1105.
Brown, B., M.D., on injections of chlorate of potassa in uterine affections, 1081.
Bruns, J. D., M.D., Essay on Life, notice of, 130.
Bruns, Victor von, treatise on practical surgery, notice of, 848.
Buchanan, Prof., methods to facilitate removal of stone from urinary bladder, 1127.
Buck, G., M.D., on post-fascial iliac abscess, 536.
Buckingham, C. E., M.D., on imperforate rectum, 151.
Bucknill, Chas., M.D., treatise on psychological medicine, review of, 826.
Bulley, on scarlatina anginosa, 1129.
Bullock's translation of Cazeaux's Midwifery, review of, 254.
Busch, Prof., notice of death of, 975.
Butcher, Mr., on excision of knee-joint, 146.
Button suture in prolapse of the vagina, 64.
in Europe, 1150.
Byrd, H. L., M.D., case of trismus nascentium, 355.
Byford, W. H., M.D., on mercurial muco-enteritis, 362.
on treatment of hydrocele, 738.

CÆCUM, perforation of, 360.
Calcareous deposits in pleura, 511.
in lungs, 515, 328.
Calcis, os, excision of, 559.
Calculi, urinary, 319, 724, 753.
of curious shape, 324, 329.
with sequestrum of bone, 754.
report of cases of, by Eve, 756.
biliary, in hepatic ducts, 324.
landing-net for, 1127.
extrusion with the fingers, of, 1127.
Calorification, physiology of, 115.
Camman, G. P., M.D., on substitutes for cod-liver oil, 1084.
Campbell, H. F., M.D., on nervous system, 129.
essays on secretory nerves, &c., review of, 222.
on traumatic tetanus, 553.
on nitrate of silver in vomiting, 1097.
Thos., M.D., case of dislocated femur reduced by, 752.
Camphor antidote to strichnia, 1099.
Cancer of pancreas, 98, 722, 883.
of liver, &c., 101.
of ileo-cæcal valve, 319.
of several organs, 323.
of a cicatrix, 330.
of omentum, stomach, &c., 512, 534.
of lung and mesenteric glands, 721, 731.
of fundus and body of uterus, 874.
escharotic treatment of, 138.
nature and treatment of, 161.
simulated by tubercle of lung, 322.
Cannabis Indica, effects of, 943.
Capillary circulation, 861.
Capsule, supra-renal, disease of, 376, 531.
Carbon, bisulphuret of, 936.
Carbonic acid gas, local use of, in diseases of uterus, 966.
Carbuncle, opiate cerate in, 1117.
Carnochan, J. M., M.D., Contributions to Surgery, notice of, 656.
Carotid artery, wound of, 159.
ligature of, 159, 350, 541, 543.
Cartwright, S. A., M.D., on hygienic treatment of phthisis, 371.
Caruncle, sinking of, after operations for strabismus, 266.
Catalepsy, nitrate of silver in, 1081.
Catalogue of Toynbee's preparations, notice of, 42.
Cataract, remarks on by Fenner, 45.
Cattle-plague, letter on by Gamgee, notice of, 35.
Cattle, evil results of over-feeding, by Gant, notice of, 846.
Cauthorn, R. S., M.D., on the virtues of asclepias Syriaca, 1090.
Cazeaux, P., treatise on midwifery by, review of, 252.
Cerebral convolutions, softening of, 506.
Cervical vertebræ, fracture of, 315.
dislocation of, 747.
Cervix uteri, laceration and severance of, 315.
engorgement of, successful treatment of, 952.
amputation of, 951.
linear écrasement of, 952.
Census of 1850, mortality statistics of, 334.

Chancre, lectures on by Ricord, review of, 606.
Chapin, Dr., on oil of tansy, 165.
Chemistry, pathological, treatise on by Becquerel and Rodier, notice of, 263.
inorganic, treatise on by Graham, notice of, 848.
Children, remitting fever of, 341.
treatise on diseases of, by Meigs, review of, 209.
by Mauthner, notice of, 505.
by Churchill, notice of, 466.
Chisholm, J. C., M.D., on tetanus, 552.
appointment of, 780.
Chlorate of potash, action of, &c., 387.
in leucorrhœa and ulcer of os uteri, 1081.
in hemorrhage, 1093.
in mercurial stomatitis, 1081.
in typhoid fever, 1088.
Chloroform in delirium tremens, 939.
in convulsions, 941.
in strychnia poisoning, 1099.
ether, antidote to, 1099.
Cholera, arsenic in, 165.
progress of, 194.
infantum, 356.
use of creasote in, 1082.
observations on, 1111.
Chorea, case presenting symptoms of, 851.
chemical composition of brain, 1104.
Christian, E. P., M.D., on therapeutical applications of electro-magnetism, 1085, 1091.
Chromic acid applied to warts, 156.
Churchill, Fleetwood, M.D., treatise on diseases of children, notice of, 466.
Cicatrix, cancer of, 330.
in the lungs, 328.
Cinchona, sulphate of, virtues of, 1090.
Cicuta in sick-headache, 946.
Circulation, physiology of, 111, 378.
Cirrhosis of liver, and bronzed skin, 377.
with diabetes, 710.
Claiborne, J. H., M.D., on treatment of remitting fever, 344.
on camphor as antidote to strychnia, 1098.
Clamp suture in prolapse of vagina, 64.
Clavicle, relations of pleura with, 119.
Climate, effects of, on tuberculous disease, 373.
Clinical lectures on medicine, by Bennett, review of, 618.
Cobb, R., M.D., on veratrum viride, 926.
Cod-liver oil in chest diseases, 162.
substitutes for, 1084.
Coffee in the Turkish fashion, 191.
antidote to many poisons, 1099.
Colby, M. F., M.D., on functions of large intestine, 131.
Colic, treatment of with antimony, 946.
Colin, M., on extirpation of pancreas, 765.
College of spirits in Paris, 972.
Collodion in orchitis, 379.
and castor oil as an artificial cuticle, 580.
Colloid cancer of omentum, 534.
Colzey, E. F., M.D., appointment of, 968.
Combe, George, M.D., necrological notice of, 1152.
Conjunctivitis, granular, use of poke-root in, 1083.
Constipation, prolonged, 1113.
Consultations with homœpaths, 779.
Contributions to operative surgery, by Carnochan, notice of, 656.
Convulsions, their pathology and treatment by Wible, 56.
puerperal, 311.
etherization in, 937.
stramonium in, 941.
Cornea, hairy mole on, 324.
Corn as an antiperiodic, 970, 1091.
Cornell, W. M., M.D., on arsenic and stillingia, 1096.
Coulson, Wm., on affections of joints in puerperal women, 581.
Coxalgia, nature and treatment of, by Gross, 658.
Coxe, E. J., M.D., on sulphate of zinc in night-sweats, 1084.
Crampton, Sir Philip, death of, 974.
Crary, D., M.D., on laryngotomy, 733.
Crawcour, J. L., M.D., on removal of warts, 156.
Creasote in dysentery and cholera infantum, 1082.
in acute cholera, 1082.
Critchett, Mr., cure of glaucoma by operation, 382.
Crosby, J., M.D., on ununited fracture, 745.
Croton oil in dropsy, 1087.
Cruveilhier, J., treatise on pathological anatomy, review of, 1.
Currey on oil of erigeron, 1093.
Cutter, B., M.D., on reduction of dislocated thumb, 749.
Cyanosis, 522.
Cystic tumors on nates of fœtus, 313.
tumor on nose, 712.
growths on intestines, 1074.
Cysts of liver expelled through lungs, 333.
expectoration of, 506.
ovarian, in child, 523.
of thyroid body, 880.
of brain, 1105.

DALTON, J. C., M.D., on bile, 128.
Daret, H., M.D., on aneurism of crural artery, 550.
Davis, R., on imperforate anus, 575.
Deadrick, Dr. W. H., necrological notice of, 205.
Deaf and dumb, observations on, 1037.
Deformity of neck of thigh-bone, 316.
Delirium tremens, shower bath in, 960.
chloroform in, 939.
Detection of poisons, treatise on by Otto, notice of, 264.
Dethan, Dr., on the use of chlorate of potash, 387.
Diagnosis, medical, treatise on, by Barclay, review of, 401.
Diarrhœa, nitrate of silver in, 504.
Dickson, Dr. Saml. H., appointment of, 588.
Dictionary of medical terms by Dunglison, notice of, 44.
Digestion, contributions to physiology of, 109.
Dilatation of heart, 521, 867.
Diseases of children, treatise on by Meigs, review of, 209.
by Churchill, notice of, 466.
Diseased meat, effects of on human health, 35, 483.
Dislocation of long bones with reference to resection, 565.
of knee, 76.
of cervical vertebræ, 747.
of fingers and thumb, 749.
of thumb, improved method of reduction, 749.
of femur reduced by manipulation, 750.
by pulleys, 752.
of head of tibia, 752.
Dispensatory, by Wood and Bache, notice of, 465.
Draper, J. C., M.D., on red line of gums in phthisis, 364.
Dropsy, use of croton oil in, 1087.
Duchatelet-Parent, treatise on prostitution by, review of, 977.
Dugas, L. A., M.D., on fracture of neck of scapula, 738.
Dunglison, Robley, M.D., Medical Dictionary by, notice of, 44.
treatise on materia medica, &c., notice of, 44.
R. J., M.D., letter on hospitals of Paris, 81.
observations on the deaf and dumb, 1037.
Dupierris, M., M.D., reduction of dislocated femur, by, 751.
Dupree, Dr., on chlorate of potash, 1093.
Dysentery, relation of to abscess of liver, 169.
epidemic, 293, 357, 358.
creasote in, 1082.
nitrate of potash in, 1082.
cathartics in, 1086.

EAR, Toynbee's catalogue of morbid preparations of, notice of, 42.
plastic operation upon, 78.
Earle, Pliny, M.D., on paralysis of the insane, 356.
Eburnation of bone, 312.
Ecstasy, case of, 353.
Ecraseur in ovariotomy, 698.
as used in Paris, 1151.
Edibles, African, 391.
Editorial change, 398.
Electro-magnetism, therapeutical uses of, 1085, 1091.
Embolia, nature and treatment of, 415, 568.
Encephaloid of thigh, 98.
of testicle, 514.
of kidney, 524.
Endemic diseases of the South, liability of negroes to, 383.
Eneuresis cured by pareira brava, 1085.
Enteritis, mercurial, 362.
Epidemic dysentery, 293, 357.
Epidemic fever with erythematic pharyngitis, 348.
Epidemics and epizootics, relations between, 483.
Epiglottic cartilage, lesions of, 369.
Epilepsy, ligature of carotid artery for, 157.
researches on, by Brown-Séquard, review of, 222.
clinical observations on, 384.
Epizootics, original researches on, by Sutton, 483.
Erectile tumor of urethra, 509.
Ergot in hemorrhage, 1094.
of wheat as an ecbolic, 1095.
Erigeron, oil of, in hemorrhages, 1093.
Erysipelas, tobacco in, 938.
iron in, 1093.
Escharotic treatment of cancer, 138.
Ether, antidote to chloroform, 1099.
Eve, Paul F., M.D., case of laryngo-tracheotomy by, 737.
lithotomy operations by, 756.
Excision of joints, 142.
of knee-joint, 146.
of maxillæ, 557, 558.
of humerus, 555, 556.
of os calcis, 559.
of hip-joint, 560.
of ankle-joint, 580.
of bones and joints in British hospitals, 955.

Excito-secretory system of nerves, by Campbell, 129.
Exostosis of scapula, 331.
of tibia and fibula, 531.
occupying orbit and nasal cavity, 554.
Eye, injury of, 1067.
Eyeball, prominence of, after operations for strabismus, 266.

FATTY degeneration of supra-renal capsule, 530.
Fatty liver, 534.
Femoral aneurism, 544, 547, 550, 551.
Femur, deformity of neck of, 316.
Femur, excision of upper extremity for necrosis, 325.
Femur, dislocation of, reduced by manipulation, 750.
Fenner, C. S., M.D., on cataract, 45.
lithotrity by, 753.
on phytolacca decandra in granular conjunctivitis, 1083.
Fever, intermittent, 344.
yellow, 348.
remittent, 344.
epidemic with erythematic pharyngitis, 348.
Fibro-fatty tumor of abdomen, 517.
Fibrous tumor of uterus removed during labor, 527.
Fibroid degeneration of liver and kidney, 531.
Fibrous tumor of breast, 706.
Fingers, apparatus for reducing dislocations of, 749.
Fischer, Emil, M.D., foreign abstracts by, 761, 947, 1104.
Fistula of gall-bladder, 95.
Flint, Austin, Senior, M.D., report on practical medicine by, 341.
appointment of, 780.
Flint, Austin, Junior, M.D., on capillary circulation, 861.
Fœtal monstrosity, 315.
Forbes, Sir John, treatise on nature and art in the cure of disease, review of, 800.
Forearm, injury of, 1065.
Foreign body in œsophagus, 158.
in stomach and bowels, 359.
in bladder, 754.
Förster, Prof., serous sac of brain, 1105.
Fracture of skull, 75.
of humerus, 310.
of cervical vertebræ, 315, 518.
of neck of scapula, 738.
of spine, 741.
of pelvis, 742.
of neck of thigh-bone, 743.
ununited, 745, 746.
Fracture, spontaneous, in rachitis, 505.
Frazier, A., M.D., on epidemic dysentery, 357.
Frick, Chas., M.D., on cirrhosis of liver, with bronzed skin, 377.
appointment of, 586.
Fries, G., M.D., on radical treatment of hydrocele, 738.
Frost bites, treatment of, 1085.
Function of right auricle, 378.
Fungus of testicle, 103.
Fusion of medical journals, 398.

GAGE, on ioduretted glycerine, 1097.
Gallaher, Dr., on chlorate of potash in mercurial stomatitis, 1081.
Gall-bladder, fistula of, 95.
diseases of, 361.
Gall-stones impacted in hepatic ducts, 324.
Gamgee, J. S., letter on diseased meat, review of, 35.
Gangrene of bladder, 96.
with ossification of arteries, 507.
cured by pyroligneous acid, 1071.
Gant, F. J., treatise on over-feeding of cattle, notice of, 846.
Garrett, Dr. F. M., on chloroform in delirium tremens, 939.
Gastric irritation a cause of puerperal convulsions, 311.
Geddings, E., M.D., lectures on surgery, notice of, 843.
Gelseminum, properties and uses of, 931.
Gendrin, A. M., D.M., treatise on anatomy of inflammations, review of, 1.
Gentiana quinqueflora, virtues of, 1090.
Gerhardt, Dr., on thrombus of cerebral sinuses, 954.
Gibb, G. D., M.D., case of wound and ligature of carotid artery, 159.
Gibbs, O. C., M.D., case simulating chorea, 851.
on strychnia in sciatica, 936.
on cathartics in dysentery, 1086.
Glaucoma, new operation for, 382.
Glottis, spasm of, laryngotomy in, 733.
Glycerine in sore nipples, 1097.
ioduretted, preparation of, 1097.
Goodrake, Dr., on asclepias tuberosa, 945.
Gorham, Dr., on veratrum viride, 928.
Gout, rheumatic, treatise on by Adams, notice of, 850.
Graham, Thos., treatise on inorganic chemistry, notice of, 848.
Gray, Asa, M.D., botanical works by, review of, 22, 265.
Green, Horace, M.D., on veratrum viride, 914.
on lesions of epiglottis, 369.

Gregory, Dr. Wm., necrological, notice of, 783.
Gros, Dr. L., on pepsin in vomiting of pregnancy, 965.
Gross, S. D., M.D., elements of pathological anatomy, review of, 1.
invention of a new tourniquet by, 540.
essay on hip-joint disease by, 658.
on dislocation of head of tibia, 752.
removal of lead-pencil from bladder by, 754.
Gums, red line of in diagnosis of phthisis, 364.
Gunn, M., M.D., on lumbar abscess, 539.
resection of lower jaw by, 556.
case of ununited fracture by, 746.

HABERSHON, S O., M.D., treatise on disease of alimentary canal, review of, 236.
Hairy mole on cornea of ox, 324.
Hammond, W. A., M.D., on nutritive value of albumen, starch, and gum, 126.
on injection of urea into the blood, 287.
Hammer, J. M., M.D., case of intussusception, 359.
Hamilton, Frank, M.D., on aneurism, 547.
on dislocations and excisions of long bones, 565.
Handy, W. R., M.D., on veratrum, viride, 923.
Hare, Dr. Robert, necrological notice of, 782.
Harper, J. D., M.D., on scarlatina, 345.
Harrison, Dr. Robt., death of, 783.
Hartshorne, E., M.D., report on practical surgery by, 536, 732.
Haskell, B., M.D., essay on physiology of nervous system, notice of, 130.
Hasse, C. E., M.D., treatise on pathological anatomy, review of, 1.
Haughton, R. E., M.D., tracheotomy by, 736.
on veratrum viride, 929.
on podophyllum and leptandria, 1079.
Hay, Dr. W., on valerianate of ammonia, 934.
Hays, Dr., on treatment of frost bites, 1085.
Heart-clots, 100.
Heart, dilatation of, 521.
malformation of, 522.
fatty degeneration of, 1106.
Hæmoptysis, endemic, 371.
Hemorrhages, passive, chlorate of potash in, 1093.
oil of erigeron in, 1093.
Hemorrhages, passive, ergot in, 1094.
ice in, 1094.
nitric acid in, 1094.
Henry, G. R., M.D., reduction of dislocation of femur by, 751.
Hepatic abscess, 525.
Hernia, reduction of by compression, 383.
strangulated, 507.
radical cure of, 758.
hydro-cerebral, 73.
Herpin, Dr. T., observations on epilepsy by, 384.
Hewson, A., M.D., on strabismus, 266.
Hip-joint, excision of, 560.
amputation at, 567.
observations on tuberculosis of, by Gross, 658.
Hippophagy, 399.
Hird, Mr., on cancer and its treatment, 161.
Histology of nervous system, 133.
Hixon, C., M D., on luxation of knee-joint, 76.
Hobson, B., treatise on anatomy, in Chinese, notice of, 43.
Hogs, epizootic disease among, 483.
Holston, J. G. F., M.D., appointment of, 1149.
Homœopaths, consultation with, 780.
Horner, W. E., M.D., treatise on morbid anatomy, review of, 1.
Hospitals of Paris, 81.
Hullihen, Dr. S. P., biographical sketch of, 199.
Human histology, treatise on by Peaslee, review of, 260.
Humerus, fracture of, 310.
excision of, 557, 558.
Hunt, S. B., M.D., resignation of, 780.
Hunton, Dr. A., on hydrocephalus, 944.
Hutchinson, J. C., M.D., on laceration of urethra, 154.
on ligature of artery of penis, 155.
Hydrocele, diagnosis of, 381.
radical treatment of, 738.
Hydrocephalus treated with lobelia, 944.
Hydrophobic hysteria, 349.
Hygienic treatment of phthisis, 371.
Hypertrophy of prepuce, 155.

ICE in hemorrhages, 1094.
Ileo-cæcal valve, cancer of, 319.
Ileus, case of, with operation for, 176.
Iliac artery, ligature of, 547, 549, 550, 551.
Iliac fossa, abscesses of, 536.
Imperforate anus and rectum, 151, 575, 727.
Indigenous medicinal plants, 944.
Infantile apoplexy, 70.

Infant mortality in large cities, by Reese, 375.
Innominate artery, aneurism of, 511.
Insanity, treatise on, by Bucknill and Tuke, review of, 826.
influence of pregnancy and delivery upon, 1145.
Institutes of medicine, treatise on, by Paine, review of, 807.
Intestinal obstruction, 176.
Intestine, large, functions of, 131.
peristaltic motion of, 769.
Intellect, effect of apoplexy upon, 351.
Intercostal neuralgia, 355.
Intermittent fever, treatment of, 343.
nitric acid in, 1089.
asclepias Syrica in, 1090.
sulphate of cinchonia in, 1090.
gentiana quinqueflora in, 1090.
electro-magnetism in, 1090.
indian-corn meal in, 1090.
prepared chalk and vinegar in, 1092.
confection of cinchona in, 1091.
Intussusception of bowels, 359.
Iodide of potassium in syphilis, 1080.
injection of, in leucorrhœa, 1081.
in asthma, 1084.
as an antigalactic, 1116.
Iodine in ovarian tumors, 1082.
in neuralgia, 1097.
Iron in erysipelas, 1093.
Isaacs, C. E., M.D., on extent of pleuræ above clavicles, 119.
on structure and functions of kidney, 120.

JACUBOWITSCH, M., on histology of nervous system, 133.
Jameson, Dr. R. H., on veratrum viride, 915.
Jarvis, Dr. E., on mortality statistics of U. S. census, review of, 334.
Jauncy, W., on lupuline, 570.
Jenner, monument to, 192, 781, 974.
Jessamine, yellow, medicinal virtues of, 931.
Jewell, W., M.D., on mortality of Philadelphia, 86.
Johnson, J. G., on fractures of neck of femur, 744.
W. J., M.D., on epidemic dysentery, 293.
Johnston, Dr. C., on tracheotomy, 735.
Joints, excision of, 142, 955.
affections of in puerperal women, 581.
Jones, Dr. Joseph, appointment of, 586.
Journal, American Veterinary, notice of, 586.
Journal of Physiology, by Brown-Séquard, notice of, 586.
Jurisprudence, obstetric, 1149.

KERR, J. G., M.D., on bony tumor of scrotum, 79.
Kidney, structure and functions of, 120.
tubercle of, 517, 868.
suppuration of, 518.
encephaloid of, 524.
fibroid degeneration of, 531, 727.
single, 715.
King, W. R., M.D., on nitrate of silver in catalepsy, 1081.
Kinloch, R. A., M.D., on excision of hip-joint, 560.
Kluge, J. P., M.D., on medullary sarcoma, 307.
Knee-joint, excision of, 146.
dislocation of, 76, 752.
Knight, Dr. N. J., on etherization in convulsions, 937.

LACERATION of urethra, 154.
of cervix uteri, 315.
Landell, R., M.D., vaccine in smallpox, 1102.
Landing-net for calculi, 1128.
Langenschwartz, Dr., on antidote to many poisons, 1099.
Laryngotomy, 733.
Laryngo-tracheotomy, 737.
Larynx, introduction of probang into, 78.
piece of beef in, 735.
ulceration of, 868, 872.
Laudanum dressings in affections of uterus, 967.
Lawson, L. M., M.D., on precursory stage of phthisis, 366.
Lebert, Prof., on pulmonary tuberculosis, 1107.
observations on cholera by, 1111.
Lectures on diseases of women, by West, review of, 594.
on chancre, by Ricord, review of, 606.
clinical, on medicine, by Bennett, review of, 618.
on principles and practice of physic, by Watson, notice of, 653.
Lee, E., on effects of climate in tuberculous disease, 373.
Lenhossek, Prof., on structure of nervous centres, 136.
Lente, F. D., M.D., on medication of larynx and trachea, 78.
on fracture of spine, 741.
Leptandria, virtues of, 1079.
Letherman, W. H., M.D., on virtues of may-apple, 1086.
Leucorrhœa, iodide of potassium in, 1081.
chlorate of potassa in, 1081.
Levergood, J. M., M.D., on puerperal convulsions, 311.
Levezey, Dr. H., on veratrum viride, 916.

Levis, R. J., M.D., on reduction of dislocation of fingers, 749, 753.
Life, essay on, by Bruns, notice of, 130.
Ligation of arteries, 155, 157, 159, 350, 541, 543, 547, 549, 550, 551.
Lithotomy operations, 754, 756.
Lithotrity, 753.
Liver, fatty degeneration of, 93.
 cancer of, 101, 731.
 cysts of, 333.
 cirrhosis of, with bronzed skin, 377.
  with diabetes, 710.
 medullary sarcoma of, 307.
 enlargement of, 514.
 abscess of, 525.
 fibroid degeneration of, 531, 868.
 fatty degeneration of, 534.
 tubercle of, 1076.
 acute yellow atrophy of, 1108.
 nutmeg, 1110.
Lobelia in hydrocephalus, 944.
Lopez, A., M.D., injury of forearm, 1066.
 injury of eye, 1067.
 physiological surgery after amputation, 1069.
 incised wound of abdomen, entering intestine, 1070.
 muscular contusion and disruption of fibres, 1070.
 gangrene cured by pyroligneous acid, 1071.
Lossetti, Dr., on collodion in orchitis, 379.
Lumbar abscess, 539.
Lungs, apoplexy of, 317.
 tubercle of, 711.
 tubercle of simulating cancer, 322.
 cancer of, 721.
 cicatrix and chalky deposits in, 328, 515.
 cyst of, 506.
Lupuline, action of, 570.

MAISONNEUVE, M., new method of amputation, by 947.
Malformation of heart, 522.
Malpighian bodies, function of, 120.
Mal-practice, suits for, 195, 389.
Malignant tumor of breast, 580.
Mammary gland, fibrous tumor of, 706.
Manipulation, reduction of dislocations by, 750.
Manual of detection of poisons, by Otto, notice of, 264.
Marsden, Mr., on secretion of urinary constituents from skin, 174.
Materia medica, report on, by Bell, 910, 1079.
Mauthner, L. W., M.D., nitrate of silver in serous diarrhœa; ass's milk in atrophy of bones; spontaneous fractures in rachitis, 504.
Mauthner, L. W., M.D., necrological notice of, 783.
Maxillæ, resections of, 555, 556.
Maxson, E. R., M.D., on dislocation of cervical vertebræ, 747.
Mayes, Dr. J. A., on yellow jessamine, 931.
Mayer, Dr. C., on amputation of cervix uteri, 951.
McAlmont, C., M.D., on hæmoptysis, 367.
McCaw, J. B., M.D., strychnia in sick-headache, 946.
 appointment of, 1149.
McCreedy, B. W., M.D., on effects of apoplexy on intellect, 351.
McClellan, Dr. J. H. B., appointment of, 968.
McElbright, T. G., M.D., reduction of dislocation of femur by, 751.
McGugin, D. L., M.D., on hydrophobic hysteria, 349.
 on veratrum viride, 924.
 on ergot of wheat, 1095.
McMate, W. H., M.D., on virtues of creasote, 1082.
Meat, diseased, letter on, by Gamgee, notice of, 35.
Meckley, J., M.D., case of fracture of humerus by, 310.
Medical students in U. S. in 1857, 585.
Medical diagnosis, treatise on, by Barclay, review of, 401.
Medicine, clinical lectures on, by Bennett, review of, 618.
 institutes of, treatise on, by Paine, review of, 807.
 psychological, treatise on, by Bucknill and Tuke, review of, 826.
Medullary sarcoma, case of, 307.
Meigs, J. F., M.D., treatise on diseases of children by, review of, 209.
Meissner, Dr., on operative midwifery, 1142.
Meningitis, with tubercle in lungs, 706.
Meningeal artery, dilatation of, 726.
Menstruation during pregnancy, 965.
Mercurial muco-enteritis, 362.
Mercier, A., M.D., on ligature of iliac artery, 550, 551.
Merei, Dr. Schœpf, necrological notice of, 591.
Mesentric glands, cancer of, 721, 731.
Metamorphosis of tissues, 114.
Metcalfe, Saml., M.D., necrological notice of, 206.
Meyer, Dr., on temperature in patients, 1116.
Midwifery, treatise on, by Cazeux, review of, 252.
 by Miller, review of, 437.
 statistics of operative, 1142.

Milk, suppression of by belladonna, 946.
Miller, H., M.D., treatise on obstetrics by, review of, 437.
resignation of, 585.
Mineral springs, 186.
Minturn, Edward, M.D., necrological notice of, 1153.
Mitchell, S. W., M.D., report on anatomy and physiology by, 105.
J. K., M.D., necrological notice of, 588.
Dr. F., case of popliteal aneurism by, 545.
Monstrosity, fœtal, 315, 397.
Morbus coxarius, nature and treatment of, by Gross, 658.
excision of hip-joint in, 560.
Morris, Caspar, M.D., essay on scarlet fever by, review of, 1010.
Morrogh, C., M.D., on excision of os calcis, 559.
Mortality of Philadelphia, 86.
Mortality statistics of U. S. census, by Jarvis, review of, 334.
Mott, A. B., M.D., on removal of exostosis, 554.
Mühlig, Prof., on yellow atrophy of liver, 1108.
Müller, Johannes, M.D., necrological notice of, 783.
M., chemical composition of brain, 1104.
Murphy, Dr. C.H., on veratrum viride, 921.
Muscular motion, essay on, by Watters, notice of, 130.
contusion, with disruption of fibres, 1070.
Mussey, W. H., M.D., on resection of maxillæ, 555.
R. D., M.D., on fractures of neck of femur, 743.

NASAL CAVITIES, removal of exostosis from, 554.
Nates, cystic tumors of, 313.
Nature and art in the cure of disease, treatise on, by Forbes, review of, 800.
Naval medical corps, appointments to, 970.
Necrosis of upper extremity of femur, 325.
Negroes, liability of, to endemic diseases of South, 383.
Nerves, excito-secretory system of, essay on by Campbell, review of, 129, 222.
Nervous system, structure of, 133, 136.
effect of red blood upon, 135.
contributions to physiology of, by Brown-Séquard, 107.
Nervous centres of uterus, 767.
Neuralgia, valerianate of ammonia in, 570.
Neuralgia, intercostal, 355.
iodine in, 1097.
Newson, Dr. B. T., on veratrum viride, 929.
New York State Inebriate Asylum, 1149.
Nichols, A. W., M.D., contributions to pathology, &c., by, 343, 353, 355, 374.
Nitrate of silver in diarrhœa, 504.
in catalepsy, 1081.
in vomiting in pregnancy, 1097.
in oxyuris vermicularis, 1116.
of potash in dysentery, 1082.
Nitric acid in pertussis, 1082.
in intermittent fever, 1089
in hemorrhage, 1094.
Norton, J. C., M.D., on cicuta in sick-headache, 946.
Norwood, Dr. W. C., on veratrum viride, 929.
Nott, Dr. J. C., resignation of, 780.
on acclimation of negroes, 383.
Nutmeg liver, 1110.
Nutrition, growth, &c., contributions to physiology of, 114.
Nutritive value of albumen, starch, and gum, by Hammond, 126.

OBSERVATIONS on the deaf and dumb, 1037.
on cholera, 1111.
on temperature of patients, 1114, 1116.
Obstetrics, treatise on, by Miller, review of, 436.
by Cazeaux, review of, 252.
O'Connor, Dr., on valerianate of ammonia, 570.
Œsophagus, foreign body in, 158.
Oil, cod-liver, in chest diseases, 162.
of tansy, toxicologically considered, 165.
Omentum, cancer of, 512, 534.
Opiate cerate in carbuncle, 1117.
Orbit, removal of exostosis from, 554.
Orchitis treated with collodion, 379.
Osgood, Dr. C., on veratrum viride, 914.
Otto, A. W., M.D., treatise on morbid anatomy by, review of, 1.
Dr. Fr. Jul., treatise on detection of poisons by, notice of, 264.
Ovarian cyst in an infant, 523.
tumors, treated by iodine, 1082.
Ovary, abscess of, 705.
Ovary, extirpation of, 698.
Oxyuris vermicularis, nitrate of silver in, 1116.

PÆONIA officinalis, virtues of, 944.
Paine, Martyn, M.D., treatise on institutes of medicine by, review of, 807.
Pancreas, cancer of, 98, 722, 883.

Pancreas, enlargement and induration of, 332, 515.
extirpation of, 765.
function of, 379.
Pancoast, Joseph, M.D., plastic operation by, 78.
Paracentesis in pleurisy, 368.
Paralysis of the insane, 356.
recovery from, 355.
Pareira brava in enuresis, 1085.
Parker, E. H., M.D., on cholera infantum, 356.
W., M.D., on iron in erysipelas, 1093.
Parotid gland, extirpation of, 732.
Pathological Society of Philadelphia, proceedings of the, 93, 312, 506, 705, 867, 1072, 1152.
Pathology *versus* phrenology, 196.
Pathology, treatise on by Bamberger, review of, 414.
Paton, G., M.D., on the perceptive power of the spinal cord, 467, 703.
Peaslee, E. R., M.D., treatise on histology by, review of, 260.
Pelvis, fracture of, 742.
Pepper, W., M.D., on diseases of gall-bladder, 361.
Pepsin in vomiting of pregnancy, 965.
Perceptive power of spinal cord, 467, 703.
Perforation of cæcum and appendix, 360.
of aortic valves, 872.
Pericardium, adhesion of, with dilatation of heart, 867.
Pericarditis, 1106.
Peristaltic action of intestines, 769.
Peritoneum, tubercle of, 1076.
Pertussis, nitric acid in, 1082.
Pharyngitis, epidemic, 348.
Philadelphia, mortality of, 92.
Phillips, D. B., M.D., on corn as an antiperiodic, 970, 1091.
H. L., M.D., on strychnia poisoning, 1099.
Phthisis, influence of hygiene upon, 371.
precursory stage of, 366.
red line of gums in diagnosis of, 364.
novel cure for, 972.
Physician's visiting list for 1859, notice of, 1037.
Physiological properties and uses of blood, 761.
surgery after amputation, 1069.
Physiology and Pathology, researches in, by Brown-Séquard, review of, 222.
journal of, by Brown-Séquard, notice of, 586.
Phytolacca decandra in granular conjunctivitis, 1083.
Pirrie on puerperal convulsions, 1138.
Plastic operation upon ear, 78.
Playfair, Dr. Lyon, appointment of, 971.
Pleuræ, extent of above clavicles, 119.
calcareous deposits in, 511.
Pleurisy, paracentesis in, 368.
Pleurisy root, properties of, 945.
Pneumonia, treatment of, 364.
by quinine, 1092.
at Belleveu hospital, 363.
Pneumothorax, case of, 374.
Podophyllum peltatum, virtues of, 1079, 1086.
Poisons, treatise on detection of, by Otto, notice of, 264.
Polypus, uterine, removed by écraseur, 160.
Popliteal aneurism, 545, 546, 579.
Post-fascial abscess of iliac fossa, 536.
Potton, Dr., treatise on prostitution by, review of, 978.
Powell, R. D., M.D., on treatment of yellow fever, 348.
Practical surgery, treatise on by Von Bruns, notice of, 848.
Practice of medicine, treatise on by Watson, notice of, 653.
by Wood, notice of, 1036.
by Tanner, notice of, 1036.
Pregnancy, influence of on development of tubercle, 372.
influence of on insanity, 1145.
Prizes at the Académie de Médecine, 398.
Probang, introduction of into larynx, 78.
Prominence of eyeball after operations for strabismus, 266.
Prolapse of vagina, 64.
Prolapsus uteri, amputation of cervix in, 951.
Prostitution, treatise on by Duchatelet, review of, 977.
by Acton, review of, 977.
by a physician, review of, 977.
by Potton, review of, 978.
Psychological medicine, treatise on by Bucknill and Tuke, review of, 826.
Puerperal convulsions, 311, 1138.
Puerperal women, affections of joints in, 581.
Pulmonary tuberculosis, 1107.
Pyæmia, 581, 770.
Pylorus, cancer of, 512.
Pyroligneous acid in gangrene, 1071.

QUACK advertisements in religious newspapers, 179.
Quarantine convention, 399.
Quinine in asthenic pneumonia, 1092.

RACHITES, spontaneous fracture in, 505.
Rattlesnake, antidote to poison of, 385, 1100.
Read, Ezra, M.D., on mortality statistics, 334.

Read, Wm., M.D., on tartrate of antimony, 1096.
Rectum, imperforate, 151, 727.
Reese, D. M., M.D., on infant mortality, 375.
Reid, D. W., M.D., treatise on ventilation by, notice of, 1035.
Remitting fever of children, 341.
  treatment of, 344.
Report on anatomy and physiology, by Mitchell, 105.
  on practical medicine, by Flint, 341.
  on practical surgery, by Hartshorne, 536, 732.
  on materia medica and therapeutics, by Bell, 910, 1079.
Reproduction, contributions to physiology of, 116.
Researches in physiology and pathology, by Brown-Séquard, review of, 222.
Resection of maxillæ, 555, 556.
  of humerus, 557, 558.
  of femur, 560.
  of long bones after dislocation, 556.
Respiration, contributions to physiology of, 112.
Retina, syphilitic inflammation of, 380, 708.
  apoplexy of, 731.
Rezzonico, Dr., on nutmeg liver, 1110.
Rheumatic gout, treatise on by Adams, notice of, 850.
  arthritis, cases of, 713.
Rice, David, M.D., on foreign bodies in œsophagus, 158.
Richardson, Dr. T. G., appointment of, 782.
Ricord's lectures on chancre, review of, 606.
Riggs, J. W., M.D., on radical treatment of hernia, 758.
Roemaer, Dr., on iodine in ovarian tumors, 1082.
Rokitansky, Carl, treatise on morbid anatomy by, review of, 1.
Rousset, Prof., on iodide of potassium as an antigalactic, 1116.
Royle, Dr. Forbes, necrological notice of, 591.
Rupture of uterus, 1137.

SALTER, Hyde, M.D., on asthma, 1134.
Sarkin, 1104.
Saunders, D.D., M.D., on red line of gums in phthisis, 364.
Savage, Dr., removal of uterine polypus by, 160.
Secretion, contributions to physiology of, 112.
Secretory and excito-secretory nerves, essays on by Campbell, review of, 222.
Sédillot, Prof., on scooping of bones, 948.
Scarlatina, treatment of, 345.
  anginosa, 1129.
Scarlet fever, treatise on by Morris, review of, 1010.
  acetic acid in, 1088.
Scapula, exostosis of, 331.
  fracture of neck of, 738.
Schenck, B. F., M.D., on acetic acid in scarlatina, 1088.
Schiell, Dr. Jas., on bisulphuret of carbon, 936.
Schultz, Dr., on nitrate of silver in worms, 1116.
Schutzenberger, Prof., on emboli, 568.
Sciatica, strychnine in, 936.
Scooping of bones in caries, &c., 948.
Scrotum, bony tumor of, 79, 100.
Shapter, Thos., M.D., on functions of right auricle, 378.
Shelby Medical College, organization of, 781.
Shoulder-joint, case of obscure injury of, 104.
Shower-bath in delirium tremens, 960.
Sigmund, Dr., on syphilis of bones and cartilages, 949.
Silver sutures in surgery, discourse on by Sims, review of, 635.
Sims, J. Marion, M.D., discourse on silver sutures by, review of, 635.
Skin, plates of diseases of, by Wilson, notice of, 466.
Skull, fracture of, 75.
Slater, Dr., case of pebble in trachea by, 734.
Smallpox cured by vaccine matter, 1102.
Smith, Dr. E., on cod-liver oil, 162.
  J. L., M.D., on remitting fever, 341.
Snow, Dr. John, necrological notice of, 975.
Special senses, contributions to physiology of, 108.
Spiegelberg, Dr. O., on nervous centres of uterus, 767.
  on peristaltic action of bowels, 769.
Spina bifida, treatment of, 1117-1127.
Spinal cord, perceptive power of, 467, 703.
  influence of on brain, 1104.
Spine, fracture of, 741.
Springs, mineral and thermal, 186, 394.
Statistics of mortality of United States, review of, 334.
  of operative midwifery, 1142.
Stellingia in diseases of skin, 1096.
Stephenson, Dr. J. G., on tobacco in erysipelas, 938.
Stevenson, J. L., M.D., use of croton oil in dropsy, 1087.
Stomach, softening of, 102.

Stomach and intestines, treatise on diseases of, by Habershon, review of, 236.
cancer of, 512, 723.
ulcer of, treatise on, by Brinton, review of, 1028.
ulcer of, 1077.
Storer, H. R., M.D., on prolapse of vagina, 64.
on criminal abortion, 1149.
Stout, Dr. A., necrological notice of, 197
Strabismus, subconjunctival operation for, 266.
Stramonium in convulsions, 941.
Strangulated hernia, 507.
Strecker, Dr., on sarkin, 1104.
Strong, P. H., M.D., on relation of anal fistula to phthisis, 371.
Strychnia in sciatica, 936.
in sick headache, 946.
antidote to, 1098, 1099, 1102.
Subjects in Vienna, 195.
Suits for mal-practice, 195, 389.
Sulphate of morphia in strychnia poisoning, 1099.
Supra-renal capsules, diseases of, 376, 530.
Surgery, report on by Hartshorne, 536, 732.
contributions to by Carnochan, notice of, 656.
lectures on by Geddings, notice of, 843.
manual of by Von Bruns, notice of, 848.
physiological after amputation, 1069.
Surgical cases, by A. Lopez, M.D., 1065.
Sutton, G., M.D., on epizootics, 483.
Swine, epizootic disease among, 483.
Syme, Prof. Jas., on escharotic treatment of cancer, 138.
Syphilis, primary, treatment of, 380.
of bones and cartilages, hereditary, 749.
iodide of potassium in, 1080.
Syphilitic retinitis, 380, 708.
ulceration of larynx, 872.

TALIAFERRO, Dr., on veratrum viride, 918.
on chlorate of potash in typhoid fever, 1088.
Tanner, T. H., M.D., manual of practical medicine by, notice of, 1036.
Tannic acid in sore nipples, 1097.
Tansy, oil of, poisonous properties of, 165.
Tartrate of antimony in colic, 945.
Temperature in patients, 1114.
Tessier, Prof., on prolonged constipation, 1113.
Testicle, fungus of, 103, 514.
Tetanus, traumatic, 552, 553.
Thermal springs, 188, 394.
Thigh-bone, fracture of neck of, 743.
Thomas, Prof. R. K., resignation of, 586.
Thompson, J. C., M.D., on nitric acid in intermittent fever, 1089.
Thornton, W. G., M.D., iodine in neuralgia, 1097.
Thrombosis, nature and treatment of, 414.
of cerebral sinuses, 954.
Thumb, new method of reducing dislocation of, 749.
Thyroid body, cysts of, 880.
Tibia, exostosis of, 531.
dislocation of head of, 752.
Tiedeman, H., M.D., on dysentery, 358, 1083.
Tobacco in erysipelas, 938.
Tonsils, excision of, 732.
Tourniquet, new form of by Gross, 540.
Toynbee, J., catalogue of preparations, notice of, 42.
Trachea, medication of, 78.
foreign body in, tracheotomy, 734, 735, 736, 737.
Travers, Mr., necrological notice of, 590.
Trephining followed by apoplexy, 320.
Trismus nascentium, recovery from, 355.
Tricuspid valve, insufficiency of, 521.
Triple births, 969.
Tubercle of lung simulating cancer, 322.
influence of pregnancy upon, 372.
of kidney, 517.
of peritonium and liver, 1076.
influence of climate upon development of, 373.
Tuberculosis pulmonary, 1107.
Tuke, D. H., M.D., treatise on psycological medicine, review of, 826.
Tumor, bony, of scrotum, 79.
encephaloid, of thigh, 78.
cystic, on nates of fœtus, 313.
erectile, of urethra, 509.
fibro-fatty, of abdomen, 517.
fibrous, of uterus, removed during labor, 527.

ULCER of the stomach, treatise on, by Brinton, review of, 1028.
of stomach, 1077.
Ulceration, syphilitic, of larynx, 872.
Ulcers of tongue, larynx, and bronchia, 868.
Uræmic convulsions of pregnancy, &c., treatise on, by Braun, review of, 1020.
Urea, injection of into blood, 287.
Urethra, laceration of from muscular contraction, 154.
erectile tumor of, 509.
Urinary calculus, 319, 324, 329, 725.

Urinary deposits, treatise on by Bird, review of, 785.
Uterus, polypus of removed by écraseur, 160.
severance of neck of, during labor, 315.
fibrous tumor of, removed during labor, 527.
nervous centres of, 767.
cancer of fundus and body of, 874.
amputation of neck of in prolapsus, 951.
linear écrasement of neck of, 952.
chloro-iodide of mercury in engorgement of neck of, 952.
carbonic acid douche in diseases of, 966.
laudanum dressings in painful diseases of, 967.
chlorate of potash injections in ulcerations of os uteri, 1081.
rupture of, new symptom of, 1137.

VACCINE matter in smallpox, 1102.
Vagina, prolapse of, 64.
Valerianate of ammonia, 570, 934.
Valve, tri-cuspid, insufficiency of, 521.
Van Buren, W. H., M.D., on ligature of iliac artery, 549.
Van Tuyl, Dr. D. B., case of calculi and sequestrum in bladder, 754.
Ventilation, treatise on, by Reid, notice of, 1035.
Ventricles, communication between, 522.
Veratria in incontinence of urine, 937.
Veratrum viride, medicinal virtues of, 914.
in aneurism, 544.
Vertebræ, cervical, fracture of, 315, 518.
Veterinary Journal, notice of, 586.
Virchow on thrombosis and embolia, 414.
on pericarditis and acute fatty degeneration of heart, 1106.
Vogel, J., M.D., treatise on morbid anatomy by, review of, 1.
Vomiting of pregnancy, pepsin in, 965.
nitrate of silver in, 1097.
Von Gutzeil, Dr., on carbuncle, 1117.

WALLACE, E., M.D., on fracture of skull with wound of brain, 75.
Walker, Dr. W. G., on veratrum viride, 919.
Walther, Dr., necrological notice of, 591.
Waring, T. S., M.D., editor of Geddings's lectures on surgery, 843.
Warts, removal of by chromic acid, 156.
Watson, Thos., M.D., treatise on principles and practice of physic, notice of, 653.
Dr., on veratrum viride, 921.
Watters, J. H., M.D., essay on muscular motion by, notice of, 130.
Wedl, Carl, M.D., treatise on morbid anatomy by, review of, 1.
West, Chas., M.D., treatise on diseases of women by, review of, 593.
Whitaker, J., M.D., case of fracture of pelvis during pregnancy, 742.
Wible, B. M., M.D., on convulsions, 56.
Wilson's plates of diseases of skin, notice of, 466.
Witsell, C., M.D., on nitric acid in pertussis, 1082.
Wood, G. B., M.D., Dispensatory by, notice of, 465.
treatise on practice of medicine by, notice of, 1036.
Jas. R., M.D., on ligature of carotid artery, 541.
E. P., M.D., on the use of gentiana quinqueflora, 1090.
Wunderlich, Prof., on temperature in patients, 1114.
Wythes, Joseph H., M.D., on infantile apoplexy, 70.

YELLOW fever, treatment of at Charity Hospital, 348.

ZINC, iodide of, in conjunctivitis, 1097.
oxide of, in night-sweats, 1084.
sulphate of, " " 1084.

